# Identifying Topics and Evolutionary Trends of Literature on Brain Metastases Using Latent Dirichlet Allocation

**DOI:** 10.3389/fmolb.2022.858577

**Published:** 2022-06-02

**Authors:** Jiarong Chen, Matt Williams, Yanming Huang, Shijing Si

**Affiliations:** ^1^ Clinical Experimental Center, Jiangmen Key Laboratory of Clinical Biobanks and Translational Research, Jiangmen Central Hospital, Jiangmen, China; ^2^ Department of Oncology, Jiangmen Central Hospital, Jiangmen, China; ^3^ Computational Oncology Group, Department of Surgery and Cancer, Imperial College London, London, United Kingdom; ^4^ Department of Radiotherapy, Charing Cross Hospital, Imperial College Healthcare NHS Trust, London, United Kingdom; ^5^ Duke University, Durham, NC, United States

**Keywords:** brain metastases, topic modelling, LDA, research trends, research topics

## Abstract

Research on brain metastases kept innovating. We aimed to illustrate what topics the research focused on and how it varied in different periods of all the studies on brain metastases with topic modelling. We used the latent Dirichlet allocation model to analyse the titles and abstracts of 50,176 articles on brain metastases retrieved from Web of Science, Embase and MEDLINE. We further stratified the articles to find out the topic trends of different periods. Our study identified that a rising number of studies on brain metastases were published in recent decades at a higher rate than all cancer articles. Overall, the major themes focused on treatment and histopathology. Radiotherapy took over the first and third places in the top 20 topics. Since the 2010’s, increasing attention concerned about gene mutations. Targeted therapy was a popular topic of brain metastases research after 2020.

## 1 Introduction

Brain metastases are a common and devastating complication of cancer. It is estimated that brain metastases develop in 20% of patients with cancer (Nayak et al., 2012; Tabouret et al., 2012) although the true rate, as measured in autopsy studies may be as high as 40% (Percy et al., 1972; Tsukada et al., 1983; Achrol et al., 2019). The prognosis of patients who develop brain metastases is poor, with only 7% surviving more than 2 years (Hall et al., 2000).

Brain metastases are the result of haematogenous seeding of spread cells from primary tumours to the brain (Achrol et al., 2019). The most common primary tumours for patients with brain metastases are lung, breast, colorectal cancers, melanoma and renal cell carcinoma (Ostrom et al., 2018; Achrol et al., 2019). Established treatments for brain metastases include surgery, chemotherapy and radiotherapy, while newer approaches include immunotherapy and targeted therapies (Soliman et al., 2016; Niranjan et al., 2019; Galldiks et al., 2020). Prognostic factors, including age, Karnofsky performance status (KPS) and control of primary tumour are well recognized, and predict median overall survival periods of between 2.3 and 7.1 months (Gaspar et al., 1997). Given their frequency and poor outcomes, there has been a substantial amount of research into identifying the mechanisms behind brain metastases and improving treatment strategies. Molecular analyses have revealed some genes specific to the risk of developing brain metastases, such as the tumour suppressor LKB1 and KRAS (Zhao et al., 2014). Gene expression profiling of brain metastases suggests metastases evolve from primary tumours in order to gain more neuronal cell characteristics and adapt to the microenvironment in the brain (Park et al., 2011; Brastianos et al., 2015).

An important element of conducting research is to understand the current literature. One way of doing this is through systematic reviews and meta-analysis. However, such approaches have very carefully defined inclusion criteria, and thus offer very detailed analysis, but only of a small portion of the literature. For example, our current systematic review and network meta-analysis of first-line treatment for brain metastases includes only randomized trials of different treatment approaches (Williams et al., 2018); it therefore explicitly excludes published work on risk factors, biology, prognosis, etc. As a consequence, such systematic reviews ignore much of the published literature (Kozlowski et al., 2021), and thus do not help us understand the literature as a whole.

One approach to obtaining a better overview of the total scope of the literature is topic modelling, and the commonest approach is latent Dirichlet allocation (LDA). LDA is a popular topic modelling algorithm that has been widely used in different areas such as marketing, economics and bioinformatics (Blei et al., 2003; Shirota et al., 2014; Liu et al., 2016; Amado et al., 2018; Kozlowski et al., 2021) and helps discover topics in large corpora of text through clustering. Rather than considering the meaning of the sentences, the LDA model breaks the input text into single words and looks at groups of words that then occur together (Delen and Crossland, 2008; Liu et al., 2016). Such an approach requires some degree of pre-processing, in terms of removing common, non-significant words, and aligning related words that may have different ending (lemmatization). LDA allows us to identify research topics across a large body of literature and, importantly, does not require us to define a target topic defined before the analyses, and thus offers a relatively unbiased view of the literature.

In this study, we retrieved articles on cancer in general and brain metastases specifically and analysed the number of articles published, extracted topics and themes using LDA, and examined trends in these over time.

## 2 Materials and Methods

### 2.1 Publication Assessment

We used a previously developed website to identify studies published and indexed in PubMed between 1947 and 2021 (https://esperr.github.io/pubmed-by-year/). We carried out two separate searches with terms of cancer and brain metastases on the platform in June 2021 to identify relevant publications, and reported numbers in each category and proportions over time.

### 2.2 Study Cohort

We searched for relevant studies with keywords of “brain metastases” (Supplementary Table S1) without limits on time or language (Soon et al., 2014; Zheng et al., 2016). The search was conducted in three databases including Web of Science (1970–2021), Embase (1947–2021) and MEDLINE (1950–2021) in June 2021. The search results were then imported into Endnote 20 (Camelot United Kingdom Bidco Limited, United Kingdom). We identified and removed duplicates with Endnote by comparing title, author, year, journal, volume and issues.

### 2.3 Data Pre-Processing

We extracted the title and abstract of every study found in the search. We pre-processed the text using a standard approach, and in line with other work (Cheng et al., 2020; Min et al., 2020). All text were converted to lowercase and we removed double spaces, special characters, and numbers. Subsequently, we applied a list of general English stop words and general words in abstracts (such as introduction, aim, purpose, method, conclusion, and discussion) to the titles and abstracts to remove non–information-bearing words from the text. We lemmatized words using the Python package scispaCy.

### 2.4 Topic Modelling and Themes

LDA, first proposed by Blei et al. (2003) in 2003, has been widely used in the biomedical literature analysis. The process for LDA is shown as follows:

First, the Dirichlet distribution *η* and *θ* in the selection process are defined: θ with parameter *α* for word selection and *η* with parameter *β* for topic section. Second, the general process for each document W is described in the following two steps:

1) Choose θ ∼ Dir(β).
2) For each of the *n* words ω_n_:
  a) Choose a topic z_n_ ∼ Multinomial(θ).
  b) Choose a word ω_n_ from p (ω_n_|z_n_; β), a multinomial probability conditioned on the topic z_n_.

Every cleaned, lemmatized abstract and title were treated as a single entry for the model. Topic analyses were conducted using the LDA model imported from the Gensim package in Python (3.0). The LDA model ignores the order of occurrence of terms and sentence structure and so regards each entry as a “bag-of-terms.” Topics were defined as co-occurrence probability of individual words from the bag-of-terms. Following Cheng’s study (Cheng and Hung, 2018), we chose the number of topics that yields the largest perplexity score. We identified the 20 topics, and manually grouped topics into related themes.

Finally, we collected all the textual data collected from every article (i.e., title and abstracts with stop words removed) into individual words to develop the text corpus for the whole data set and subsequently analyzed the word frequency using CountVectorizer in the Python package scikit-learn.

### 2.5 Visualization of Topics

Word clouds of the top 20 topics were generated using the WordCloud package in Python. Figures were plotted independently for each topic based on the first 20 terms. Size variation of the terms indicated the probability of the term in that topic.

### 2.6 Topic Trends

To illustrate the change of research trends on brain metastases in different periods, we stratified the data into cohorts by decades of the publishing dates. We illustrated the top 10 topics with 20 terms in the analysis for each cohort.

## 3 Results

### 3.1 Publication Trends

**FIGURE 1:**
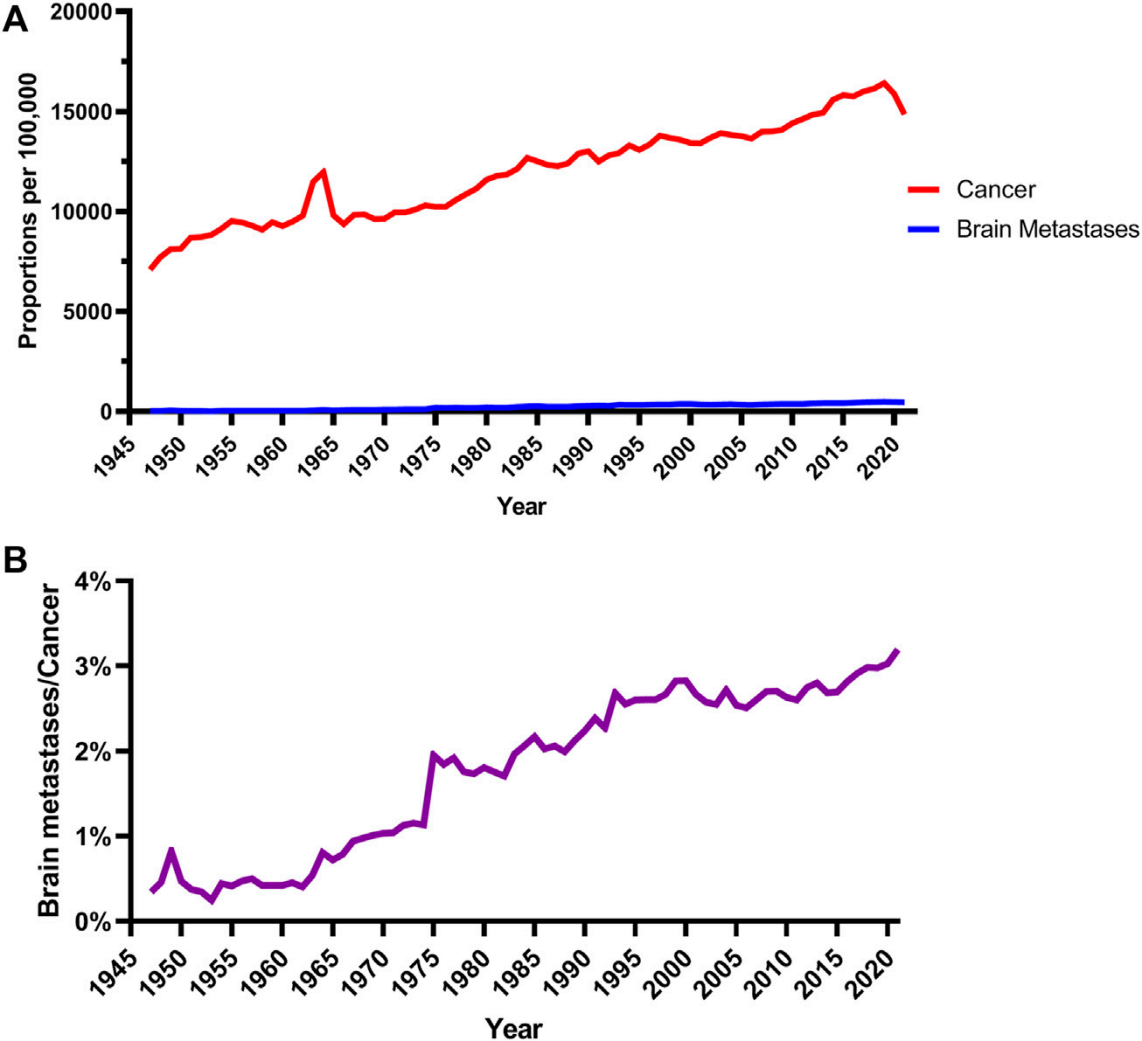
Publication trends on cancer and brain metastases. **(A)** Annual PubMed proportion for cancer and brain metastases (source: https://esperr.github.io/pubmed-by-year/). **(B)** Percentage of articles on brain metastases in all cancer studies.

Besides the absolute numbers of studies on brain metastases, it is useful to look at relative proportions. As illustrated in Figure 1A, literature on both brain metastases and cancer took up increasing proportions of all publications on PubMed during 1947 and 2021. All cancer research formed 7% of the articles in 1947, but gradually increased to 14% by 2009. However, within that general increase in research on brain metastases, brain metastases comprised less than 1% of all cancer research before 1968, but was over 3% by 2020 (Figure 1B).

### 3.2 Data Inclusion

**FIGURE 2:**
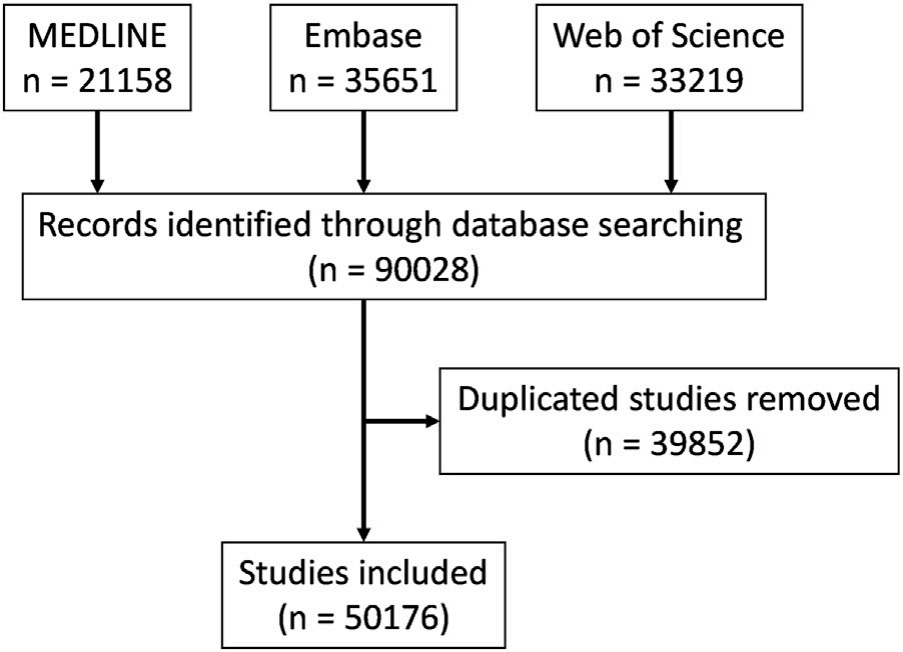
Flow chart of study inclusion.

**FIGURE 3:**
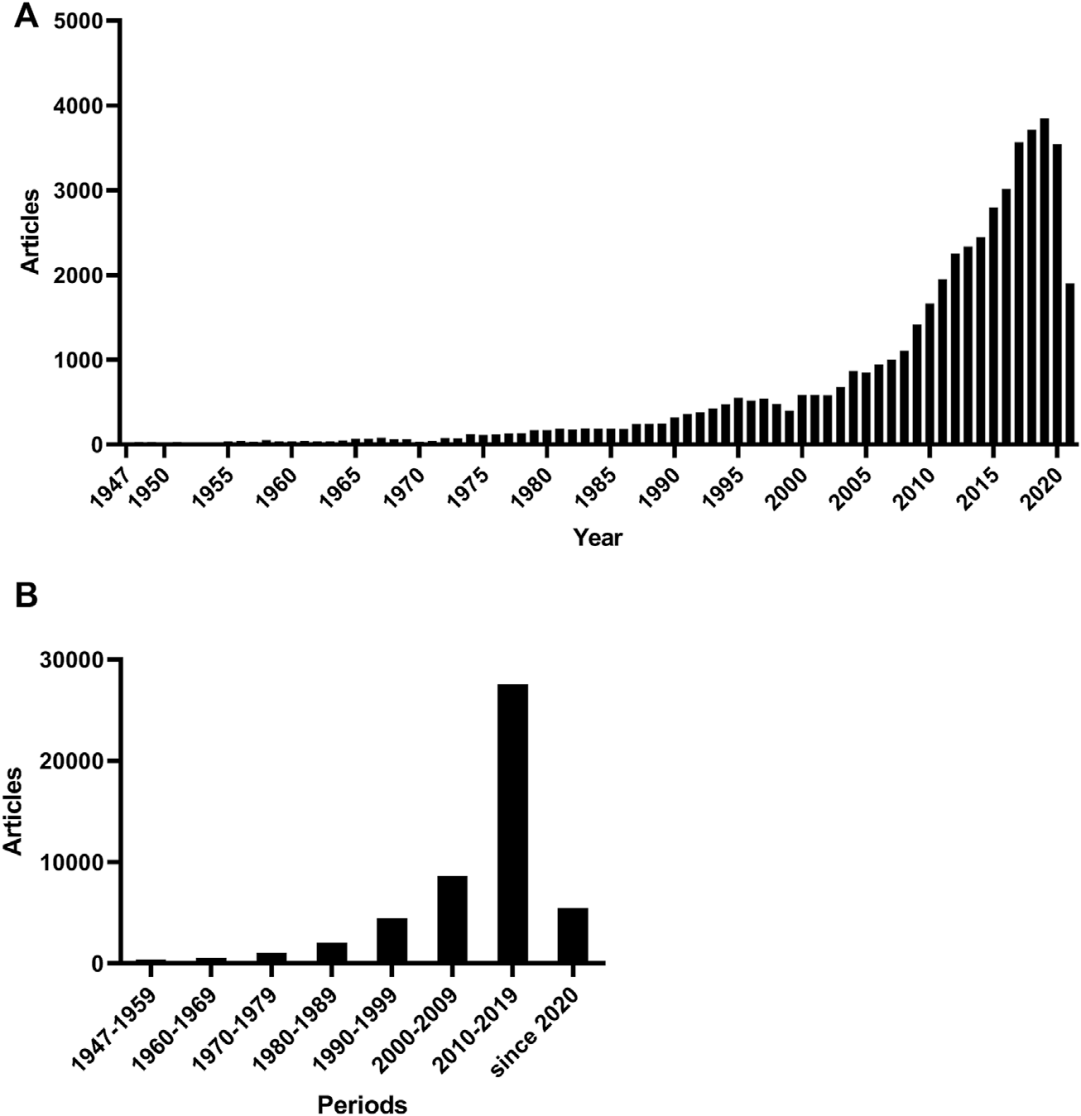
The number of articles by year of publication.

We retrieved 90,028 results, including 21,158 from MEDLINE, 35,651 from Embase and 33,219 from Web of Science. After duplicates removal, 50,176 results remained for further analysis (Figure 2). There were fewer than 50 articles on brain metastases per year before 1955 but the number kept increasing steadily between the 1960’s and 1980’s and reached 100 publications per year in 1974. The increase rate became even higher since 2000 and publications stayed above 1,000 per year after 2007 (Figure 3A). The articles published in the 2010’s overtook the total number between 1947 and 2009 (Figure 3B).

### 3.3 Topics on Brain Metastases

**FIGURE 4:**
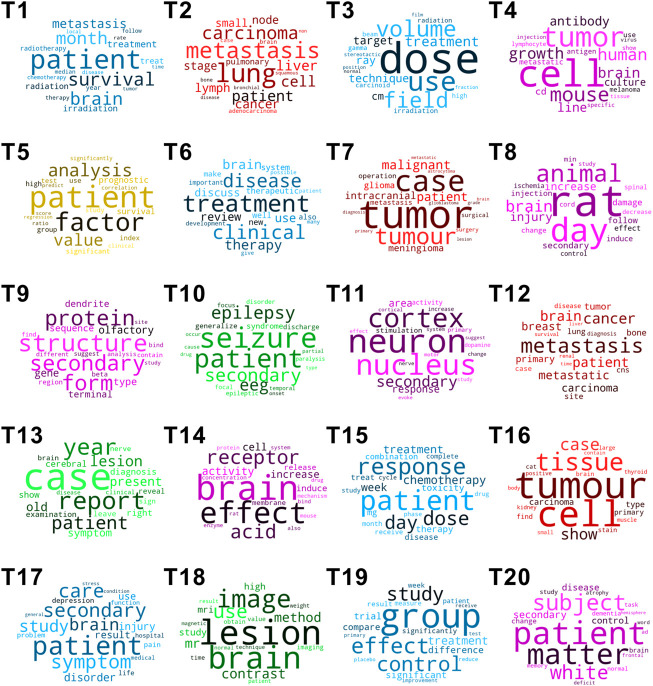
Term frequency clouds of 20 topics on brain metastases. Topics in blue: topics related to treatment (T1, T3, T6, T15, T17, and T19). Topics in red: topics related to tumour relationship (T2, T7, T12, and T16). Topics in purple: topics related to histopathology (T4, T8, T9, T11, T14, and T20). Topics in mocha: topic related to prognosis (T5). Topics in green: topics related to diagnosis (T10, T13, and T18).

We identified 20 topics for brain metastases-related articles (Table 1; Figure 4). These topics correlated with different areas, including treatment (T1, T3, T6, T15, T17, and T19), tumour relationship (T2, T7, T12, and T16), histopathology (T4, T8, T9, T11, T14, and T20), diagnosis (T10, T13, and T18) and prognosis (T5). Treatment for brain metastases was the commonest topic, and in particular radiotherapy played a key role in two leading topics (T1 and T3). Another major theme was around discovering the fundamental mechanism of brain metastases.

**TABLE 1:** The highest frequent terms for 20 topics of the brain metastases-related articles.

Topic id	Topics	Highest frequent terms of topics
1	Radiotherapy	patient, survival, brain, month, metastasis, treatment, irradiation, treat, radiation, radiotherapy, year, therapy, median, follow, chemotherapy, disease, rate, tumour, local, time
2	Lung cancer	lung, metastasis, carcinoma, patient, cell, liver, cancer, lymph, stage, node, small, pulmonary, adenocarcinoma, brain, case, bone, bronchial, squamous, non, disease
3	Stereotactic radiosurgery	dose, use, volume, field, treatment, technique, target, ray, cm, irradiation, gamma, radiation, beam, carcinoid, high, stereotactic, fraction, position, film, normal
4	Basic science	cell, tumour, mouse, human, growth, brain, antibody, line, culture, cd, metastatic, antigen, use, melanoma, lymphocyte, specific, show, virus, injection, tissue
5	Prognosis	patient, factor, analysis, value, prognostic, survival, group, significant, index, test, use, high, study, significantly, ratio, correlation, clinical, score, predict, regression
6	Treatment development	treatment, clinical, disease, therapy, brain, use, review, discuss, therapeutic, well, new, system, also, make, important, development, give, patient, possible, many
7	Primary brain tumour	tumour, case, tumour, malignant, patient, intracranial, meningioma, glioma, operation, metastasis, surgical, surgery, glioblastoma, astrocytoma, metastatic, primary, lesion, diagnosis, brain, grade
8	Brain damage research on animal	rat, day, animal, brain, injury, increase, secondary, damage, follow, injection, induce, effect, change, min, spinal, control, ischemia, cord, decrease, study
9	Protein structure	structure, secondary, protein, form, olfactory, gene, type, sequence, dendrite, terminal, region, beta, different, find, contain, analysis, bind, suggest, site, study
10	Symptoms	seizure, patient, secondary, epilepsy, eeg, syndrome, generalize, discharge, focal, focus, disorder, epileptic, paralysis, temporal, cause, onset, occur, partial, type, drug
11	Nervous system	neuron, nucleus, cortex, secondary, response, area, stimulation, activity, primary, dopamine, increase, change, effect, motor, study, cortical, nerve, system, suggest, evoke
12	Primary cancer	metastasis, cancer, brain, patient, metastatic, breast, carcinoma, primary, tumour, bone, site, lung, cns, case, disease, diagnosis, renal, survival, liver, time
13	Symptoms & lesion characteristics	case, report, year, patient, lesion, present, old, symptom, diagnosis, show, cerebral, right, examination, leave, reveal, sign, brain, nerve, clinical, disease
14	Pharmacology	brain, effect, receptor, acid, activity, increase, cell, induce, release, membrane, concentration, bind, also, enzyme, mechanism, mouse, protein, system, rat, drug
15	Chemotherapy	patient, response, dose, day, chemotherapy, treatment, week, toxicity, therapy, mg, combination, disease, treat, complete, receive, month, study, phase, drug, cycle
16	Tumour type	tumour, cell, tissue, case, show, carcinoma, type, primary, find, stain, brain, cat, large, positive, small, muscle, thyroid, kidney, body, contain
17	Palliative care	patient, secondary, care, symptom, study, brain, disorder, injury, use, result, depression, pain, problem, life, hospital, function, medical, condition, stress, general
18	Imaging	lesion, brain, image, use, contrast, method, mr, study, high, mri, imaging, value, obtain, time, weight, patient, technique, result, normal, magnetic
19	Clinical trials	group, effect, control, study, treatment, significant, trial, difference, compare, significantly, result, week, measure, patient, improvement, reduce, placebo, receive, primary, test
20	White matter and cognitive deficit	patient, matter, subject, white, secondary, control, disease, change, brain, study, dementia, normal, memory, task, atrophy, ad, word, deficit, frontal, hemisphere

### 3.4 Topic Trends in Different Periods

There were 251 studies whose publishing years were not available. Thus, these studies were excluded from the trend analysis. Given the small number of studies (*n* = 77) published between 1947 and 1949, we included studies from 1947–1949 in the 1950’s cohort (Supplementary Table S2).

During 1947–1959, most of the articles tended to be descriptions of the symptoms, primary cancer and survival of patients with brain metastases. In the 1960’s, more studies reported data on the detection of metastatic lesions from autopsy or scan. Chemotherapy became the commonest term for the first time in four of the top 10 topics in the 1970’s (T1, T5, T6, and T10). Topic trends also revealed the involvement of improving imaging techniques in diagnosing brain metastases. Computed tomography (CT) firstly occurred in the term list of the 1970’s, followed by magnetic resonance imaging (MRI) and positron emission tomography (PET) in the topic terms of the 1980’s and 1990’s. These imaging techniques remained of interest during 2000 and 2019. The involvement of new techniques in radiation also emerged since the 1990’s focusing on stereotactic radiotherapy (SRS). Starting from the 2010’s, terms related to gene mutations, such as epidermal growth factor receptor (EGFR), tyrosine kinase inhibitor (TKI) and anaplastic lymphoma kinase (ALK), became increasingly common in the articles. Targeted therapy was a popular topic of brain metastases research after 2020.

## 4 Discussion

Brain metastases occur in more than 20% of all cancer patients and carry a poor prognosis (Nayak et al., 2012; Tabouret et al., 2012). Research into brain metastases is important, and existing approaches, such as systematic reviews, while important, are limited in their scope. In this study, we retrieved articles on brain metastases and analysed the topics with the LDA model. Furthermore, we split the articles into cohorts according to their published dates and illustrated topics of different periods. An increasing number of articles on brain metastases have been published since 1950, rising at a higher rate than overall cancer research (Figure 1). We identified 20 main topics for articles and grouped these into 5 themes, of which treatment was the commonest.

We used LDA in this work as it allows us to identify research topics in the text of published studies and importantly does not require predefined target topics. In that sense, LDA allows us to develop an unbiased report of the literature, in contrast to systematic reviews which impose strict criteria. It also has convenient computational properties that allow us to scale up the analysis, and thus assess very large bodies of work.

Within the 20 different topics, we identified five themes that show the main areas of interest in publications about brain metastases. As expected, treatment was the commonest theme which accounted for six out of 20 topics, and importantly, the first and third commonest topics. Other topics were focused on the relationship between brain metastases and primary tumours, as well as histopathology, with an interest in understanding the mechanism of metastasis. Apart from these, prognosis and diagnosis were also important themes.

Radiotherapy appeared in both the first and third topics, indicating its crucial role in the treatment of brain metastases (Table 1; Figure 4). Traditionally, radiotherapy has been delivered as whole brain radiotherapy (WBRT) (Soffietti et al., 2005). However, there have been long-standing attempts to improve outcomes by varying dose and fractionation since the early 1960’s (Chu and Hilaris, 1961; Peirce, 1964; Hindo et al., 1970; Hendrickson, 1977), which correlates with the importance of radiotherapy in our data since the 1960’s (Supplementary Table S2).

The development of SRS which offers better local control and less normal tissue dosimetry (Soffietti et al., 2005; Graham et al., 2010; Abraham et al., 2018), has influenced the development of the literature. Since the 1990’s, nearly all topics which included radiotherapy focused on the use of stereotactic techniques and related topics accounted appeared in at least one of the top 10 topics in those decades (T1 in the 1990’s, T7 and T8 in the 2000’s, T3 and T9 in the 2010’s) (Supplementary Table S2).

Chemotherapy and related terms first occurred as the topmost terms in four of the top 10 topics in the 1970’s (T1, T5, T6, and T10) (Supplementary Table S2) when there were a variety of studies trying to improve the outcome of brain metastases patients with chemotherapy (Gercovich et al., 1975; Black, 1979). However, this then decreased after the 1970’s as people became aware of the effect of the blood-brain barrier in reducing the effect of chemotherapy in brain metastases. More recent work focuses on combining chemotherapy and radiotherapy (T9 in the 1990’s, T1 in the 2000’s) (Supplementary Table S2).

There has been a substantial increase in interest in the basic science associated with brain metastases since the 2000’s (T6 in the 2000’s, T2, T7, T8, and T10 in the 2010’s, T2, T8, T9, and T10 since 2020). Targetable mutations, such as EGFR and ALK, were commonly reported in the studies since the 2010’s (T2, T7, T8, and T10 in the 2010’s), along with the relevant targeted therapy agents, including crizotinib, alectinib, and lorlatinib (T4 since 2020) (Hida et al., 2017; Martínez et al., 2017; Camidge et al., 2018; Khandekar et al., 2018) (Supplementary Table S2).

Imaging plays an important role in the diagnosis and management of brain metastases, and the topic trends follow this. CT first occurred in the term list of the 1970’s (T3 in the 1970’s), followed by MRI and PET which appeared in the top 10 topics of the 1980’s (T10 in the 1980’s) and 1990’s (T1 in the 1990’s). Imaging techniques for brain metastases remained a popular topic between 2000 and 2019 (T5 and T9 in the 2000’s, T1 and T4 in the 2010’s, T5 since 2020) (Supplementary Table S2).

The major omission is surgery. Despite the key role of surgery in the management of brain metastases, especially for large metastases (Soffietti et al., 2017; Rosenfelder and Brada, 2019) and several randomized trials showing the benefits of surgery combined with WBRT (Mintz et al., 1996; Gállego Pérez-Larraya and Hildebrand, 2014), surgically associated terms such as resection occurred only in topics related to radiosurgery (T7 in the 2000’s, T3 in the 2010’s) (Supplementary Table S2). This is in keeping with a general lack of research in surgery, and is a good example where the small number of studies examining surgery is a reflection of the weakness of the literature, rather than a measure of the relative importance of surgery.

There are some limitations to this study. First, even though we did not set limitations on languages or publication time, it is difficult to include all articles especially those written not in English or published before 1947 due to the restrictions in the databases we used. Meanwhile, the number of articles in the initial period was relatively small so that we combined articles between 1947 and 1949 with those of the 1950s when analysing topics of different periods. Second, an inspection of titles and abstracts shows many recent articles used new words and terms; however, these newer topics did not occur often enough to make the top 20 topics overall. Thirdly, the LDA model breaks sentences into a package of separate words and is more likely to consider their frequency. Therefore, the results may not convey the original context and significance of some phrases.

Overall, brain metastases remain a challenging clinical problem with high morbidity and poor prognosis. We have used LDA to provide an unbiased report of all the research into brain metastases since the last 1940's, and compared it to the baseline amount of research into cancer. It is notable that the literature on brain metastases has risen to occupy a larger proportion of the published cancer literature over time, and that the main therapeutic approach that dominates the literature is radiotherapy. While we do not suggest that simple count is sufficient to measure importance (i.e. there may be a few key, practice changing trials that involve surgery or chemotherapy), it does help us understand the scope of the literature. We think that this is important for several reasons. Firstly, it helps us understand that general scope of all literature in brain metastases. Secondly, it highlights where we might to focus our efforts for systematic reviews, where there may be more literature to review. Thirdly, it highlights both where they may be options to optimize existing treatments (e.g. optimizing radiotherapy) and also to address deficits in the literature (e.g. the relative absence of literature on surgery). This is important for clinicians, and also for research funders, who may want to reflect on the potential routes to improving the areas of research conducted in brain metastases.

## 5 Conclusion

In this paper, we presented an analysis of topics on brain metastases research by utilizing LDA modelling, which revealed the history of brain metastases studies and illustrated how treatment and diagnostic techniques developed in different periods. We found that brain metastases attracted increasing attention with a higher rate than overall cancer research, especially since 2000. Among all research on brain metastases, the most common themes were treatment and histopathology and radiotherapy occupied the first and third places in the top 20 topics, demonstrating its crucial role in brain metastases research.

## Data Availability

The raw data supporting the conclusion of this article will be made available by the authors, without undue reservation.

## Abbreviations

ALK, anaplastic lymphoma kinase; CT, computed tomography; EGFR, epidermal growth factor receptor; KPS, Karnofsky performance status; LDA, latent Dirichlet allocation; MRI, magnetic resonance imaging; PET, positron emission tomography; SRS, stereotactic radiotherapy; TKI, tyrosine kinase inhibitor; WBRT, whole brain radiotherapy.
