# Supplementary material for: Identifying Topics and Evolutionary Trends of Literature on Brain Metastases Using Latent Dirichlet Allocation
Source: Front Mol Biosci. 2022 Jun 2;9:858577. doi: 10.3389/fmolb.2022.858577 (PMC9201447; doi:10.3389/fmolb.2022.858577)
Supplement: Supplementary file 1 [file Table1.DOCX]

Supplementary Material

# Supplementary Tables

**Table S1** Search strategy of brain metastases.

| **MEDLINE** |
| --- |
| ((brain OR cerebral OR intracranial OR intracerebral) adj5 (metasta* OR metastasis OR metastases)).ti,ab. |
| **EMBASE** |
| ((brain OR cerebral OR intracranial OR intracerebral) adj5 (metasta* OR metastasis OR metastases)).ti,ab. |
| **Web of Science** |
| #1 TI=(intracranial metasta* OR intracranial metastasis OR intracranial metastases)  #2 AB=(intracranial metasta* OR intracranial metastasis OR intracranial metastases)  #3 KP=(intracranial metasta* OR intracranial metastasis OR intracranial metastases)  #4 TI=(cerebral metasta* OR cerebral metastasis OR cerebral metastases)  #5 AB=(cerebral metasta* OR cerebral metastasis OR cerebral metastases)  #6 KP=(cerebral metasta* OR cerebral metastasis OR cerebral metastases)  #7 TI=(brain metasta* OR brain metastasis OR brain metastases)  #8 AB=(brain metasta* OR brain metastasis OR brain metastases)  #9 KP=(brain metasta* OR brain metastasis OR brain metastases)  #10 #1 OR #2 OR #3 OR #4 OR #5 OR #6 OR #7 OR #8 OR #9 |

**Table S2** The highest frequent terms for 10 topics of different periods on brain metastases.

| **Topic ID** | **Highest frequent terms of topics** |
| --- | --- |
| **1947-1959** | |
| 1 | case, metastasis, cancer, carcinoma, tumour, cerebral, lung, brain, patient, metastatic, show, month, right, breast, pulmonary, leave, primary, find, report, diagnosis |
| 2 | case, metastasis, tumour, cerebral, patient, metastatic, brain, show, growth, diagnosis, examination, primary, month, report, pulmonary, malignant, lesion, age, discuss, abscess |
| 3 | case, metastasis, cerebral, brain, bronchial, metastatic, carcinoma, lymph, pulmonary, lung, disease, patient, node, report, treat, show, tumour, treatment, right, yr |
| 4 | metastasis, case, tumour, primary, treatment, patient, cerebral, metastatic, cancer, present, glioblastoma, brain, year, lesion, metastasize, symptom, report, result, clinical, lung |
| 5 | metastasis, brain, tumour, cerebral, case, find, metastatic, carcinoma, patient, primary, present, diagnosis, picture, show, intracranial, symptom, follow, pulmonary, also, autopsy |
| 6 | metastasis, case, carcinoma, cerebral, patient, brain, area, metastatic, lung, tissue, clinical, primary, hypophysectomy, author, report, subcutaneous, meningeal, neoplastic, diffuse, present |
| 7 | case, metastasis, treatment, tumour, month, cancer, patient, activity, cerebral, operation, brain, area, show, hypophysectomy, high, also, week, change, effect, breast |
| 8 | case, metastasis, meningeal, primary, tumour, sugar, follow, patient, pulmonary, fluid, low, value, operation, carcinoma, right, year, intracranial, cerebral, bronchial, carcinomatosis |
| 9 | tumour, metastasis, case, cerebral, patient, intracranial, brain, carcinoma, metastatic, lung, primary, lesion, pulmonary, cell, right, study, month, report, diagnosis, breast |
| 10 | abscess, case, brain, penicillin, treatment, metastatic, lung, metastasis, pulmonary, due, early, carcinoma, cerebral, child, bronchial, treat, author, oesophagus, symptom, report |
| **1960-1969** | |
| 1 | brain, tumor, metastasis, case, patient, metastatic, scan, carcinoma, cerebral, lesion, tumour, find, study, treatment, primary, intracranial, examination, radioactive, diagnosis, present |
| 2 | case, patient, metastasis, brain, cerebral, cancer, tumor, lesion, lung, metastatic, clinical, study, diagnosis, tumour, carcinoma, primary, syndrome, report, treatment, intracranial |
| 3 | case, metastasis, brain, metastatic, report, cell, tumour, cerebral, carcinoma, patient, tumor, disease, lung, clinical, intracranial, find, malignant, primary, present, choriocarcinoma |
| 4 | metastasis, cell, cerebral, case, brain, carcinoma, cancer, patient, tumour, tumor, result, clinical, present, metastatic, treatment, bronchial, primary, show, organ, secondary |
| 5 | metastasis, brain, case, melanoma, cerebral, patient, lesion, report, year, malignant, primary, tumor, old, find, sign, organ, therapy, present, carcinoma, tumour |
| 6 | metastasis, case, brain, cerebral, carcinoma, tumor, cancer, patient, intracranial, tumour, treatment, metastatic, primary, report, find, present, show, pulmonary, lung, clinical |
| 7 | tumor, metastasis, case, patient, brain, radiation, clinical, intracranial, autopsy, cerebral, study, therapy, cell, stain, type, carcinoma, result, therapeutic, metastatic, treat |
| 8 | metastasis, patient, brain, case, tumor, report, metastatic, cerebral, cancer, tumour, intracranial, clinical, therapy, carcinoma, scan, symptom, primary, spread, radiation, liver |
| 9 | metastasis, patient, tumor, carcinoma, cerebral, case, tumour, intracranial, primary, brain, find, diagnosis, year, metastatic, report, liver, cancer, present, cell, lung |
| 10 | tumor, metastasis, malignant, case, cell, cerebral, brain, show, fluorescence, tumour, primary, finding, carcinoma, year, autopsy, intracranial, symptom, metastatic, find, great |
| **1970-1979** | |
| 1 | patient, brain, metastasis, scan, cerebral, case, tumor, study, metastatic, lesion, lung, follow, diagnosis, liver, disease, chemotherapy, primary, positive, clinical, bone |
| 2 | case, tumor, metastasis, metastatic, brain, patient, lesion, diagnosis, report, primary, cell, study, cerebral, present, therapy, glioma, use, intracranial, scan, surgical |
| 3 | brain, patient, case, metastatic, metastasis, tumor, cerebral, scan, carcinoma, intracranial, treatment, lesion, study, ct, report, therapy, primary, multiple, disease, positive |
| 4 | cerebral, metastasis, diagnosis, case, tumor, brain, method, carotid, patient, adenocarcinoma, artery, use, cancer, contrast, vascular, study, male, disease, tomography, malignant |
| 5 | tumor, brain, metastasis, cell, patient, case, treatment, type, lung, primary, study, glioma, irradiation, cerebral, show, result, metastatic, report, carcinoma, methotrexate |
| 6 | metastasis, cerebral, treatment, patient, case, carcinoma, brain, tumor, metastatic, result, cancer, therapy, chemotherapy, survival, disease, combination, malignant, lung, breast, radiation |
| 7 | brain, case, tumor, metastasis, metastatic, cerebral, patient, lesion, diagnosis, cancer, present, clinical, scan, intracranial, result, disease, report, symptom, carcinoma, primary |
| 8 | tumor, brain, cell, patient, metastasis, metastatic, tumour, normal, case, cerebral, glioma, disease, level, high, lung, tissue, study, carcinoma, show, blood |
| 9 | tumor, cell, brain, patient, metastasis, malignant, line, study, metastatic, use, treatment, rate, surgical, glioma, survival, cerebral, case, follow, increase, primary |
| 10 | patient, brain, metastasis, tumor, metastatic, treatment, therapy, diagnosis, cell, type, carcinoma, chemotherapy, disease, case, study, cancer, cerebral, lung, clinical, malignant |
| **1980-1989** | |
| 1 | pituitary, band, case, carcinoma, report, metastasis, brain, metastatic, clinical, literature, patient, cancer, primary, review, level, malignant, disable, broad, distinguish, rare |
| 2 | tumor, primary, patient, nmr, cell, brain, syndrome, trimester, malignancy, nonfunctione, case, know, encephalitis, trauma, metastasis, show, cerebral, distinguish, study, cancer |
| 3 | connective, arrangement, brain, metastasis, patient, case, isoenzyme, cell, carcinoma, metastatic, dic, interruption, lesion, association, cancer, cat, tumor, report, contribution, tumour |
| 4 | myxoma, artery, distal, cardiac, intracranial, metastasis, metastatic, branch, man, bone, cerebral, cause, patient, result, phenomenon, occur, radiographic, middle, temporal, carotid |
| 5 | patient, tumor, metastasis, brain, survival, phase, year, cell, dose, study, month, lung, treatment, primary, syndrome, radiation, metastatic, therapy, carcinoma, plasma |
| 6 | metastasis, carcinoma, patient, positive, case, compression, cell, bone, lung, cord, autonomic, cancer, cerebral, leukemia, spine, lesion, report, brain, metastatic, bromocriptine |
| 7 | metastasis, patient, brain, tumor, primary, cns, metastatic, scan, location, workup, white, melanoma, follow, compute, document, evaluation, malignant, child, bone, disease |
| 8 | carcinoid, cerebral, metastasis, deposit, tumour, predictive, intestinal, parenchymal, case, primary, unusual, cystic, brain, unknown, remain, report, distribution, clinical, course, patient |
| 9 | patient, cancer, brain, metastasis, chemotherapy, cell, lung, small, month, radiation, stage, treatment, compression, cord, disease, median, irradiation, survival, radiotherapy, develop |
| 10 | gh, mri, mammography, tumor, patient, pbl, brain, case, level, imaging, increase, pituitary, metastasis, class, metastatic, abrupt, administration, choice, phase, carcinoma |
| **1990-1999** | |
| 1 | radiosurgery, pet, stereotactic, treatment, patient, use, knife, dose, gamma, fdg, target, technique, lesion, volume, tumor, result, brain, method, metastasis, clinical |
| 2 | brain, tumor, metastasis, cell, carcinoma, case, report, year, patient, metastatic, old, leave, cd, show, month, renal, cancer, chemotherapy, lung, reveal |
| 3 | tumor, metastasis, patient, brain, metastatic, primary, intracranial, case, bone, image, carcinoma, study, cancer, show, hemorrhage, mr, mibi, lung, imaging, result |
| 4 | brain, tumor, cell, metastasis, expression, level, gene, human, high, metastatic, tissue, factor, cd, patient, mmp, normal, melanoma, study, use, increase |
| 5 | tumor, brain, metastasis, meningioma, cell, glioma, tumour, breast, study, primary, metastatic, cyst, timp, cancer, expression, case, tissue, patient, use, human |
| 6 | tumor, brain, lesion, use, meningioma, high, diagnosis, astrocytoma, cell, positive, image, patient, malignant, study, metastatic, grade, tissue, glioma, case, metastasis |
| 7 | brain, metastasis, patient, cancer, symptom, phenytoin, gastric, year, colorectal, treatment, complication, case, neurological, intracranial, therapy, clinical, disease, show, metastatic, diagnosis |
| 8 | metastasis, melanoma, patient, cerebral, scalp, nih, cell, brain, haemorrhage, malignant, case, fse, metastatic, tumor, gram, ir, human, lung, treatment, adenocarcinoma |
| 9 | patient, tumor, brain, survival, treatment, month, metastasis, chemotherapy, metastatic, treat, median, dose, year, primary, rs, therapy, follow, use, study, leptomeningeal |
| 10 | patient, tumor, metastasis, brain, treatment, local, month, therapy, cell, survival, treat, year, response, dose, lesion, control, disease, follow, metastatic, median |
| **2000-2009** | |
| 1 | patient, chemotherapy, radiotherapy, response, result, study, receive, disease, group, dose, treatment, year, rate, trial, lung, small, month, cancer, cell, survival |
| 2 | patient, brain, csf, cancer, level, cerebrospinal, metastasis, fluid, tumor, cell, serum, chemotherapy, metastatic, gpr, meningitis, hcg, disease, marker, kiss, liver |
| 3 | cell, tumor, brain, growth, mouse, human, cancer, model, expression, metastasis, metastatic, receptor, target, breast, result, increase, show, use, express, study |
| 4 | glioma, tumor, expression, grade, brain, use, tissue, cell, high, gbm, astrocytoma, human, result, glioblastoma, level, beta, plasma, low, normal, concentration |
| 5 | image, metastasis, mri, brain, mr, imaging, contrast, tumor, lesion, high, value, diffusion, perfusion, patient, weight, enhance, use, resonance, magnetic, cerebral |
| 6 | expression, cancer, cell, protein, gene, brain, prostate, signal, metastasis, metastatic, bind, identify, tissue, express, tumor, mutation, associate, adam, show, line |
| 7 | patient, metastasis, brain, survival, cancer, month, treatment, median, lung, wbrt, year, resection, treat, radiotherapy, disease, therapy, radiosurgery, breast, result, control |
| 8 | brain, patient, tumor, treatment, metastasis, use, cancer, therapy, study, result, clinical, metastatic, radiosurgery, radiation, disease, system, include, control, method, review |
| 9 | pet, ct, patient, mri, metastasis, lesion, fdg, image, scan, brain, case, lung, imaging, tomography, use, body, stage, detect, diagnosis, metastatic |
| 10 | metastasis, patient, brain, case, cancer, tumor, primary, lung, diagnosis, report, breast, lesion, metastatic, year, present, treatment, cell, clinical, disease, malignant |
| **2010-2019** | |
| 1 | tumor, brain, mri, image, high, use, metastasis, value, contrast, glioma, imaging, patient, analysis, mr, method, study, grade, lesion, result, enhance |
| 2 | patient, metastasis, egfr, survival, brain, factor, cancer, mutation, analysis, lung, prognostic, month, nsclc, associate, tki, ci, os, group, study, risk |
| 3 | patient, treatment, brain, metastasis, therapy, radiation, management, review, surgery, clinical, tumor, resection, surgical, study, stereotactic, use, cancer, radiosurgery, metastatic, disease |
| 4 | pet, ct, patient, brain, imaging, fdg, mri, lesion, scan, uptake, metastasis, image, tumor, tomography, study, detection, result, detect, use, emission |
| 5 | tumor, case, patient, cns, central, nervous, leptomeningeal, system, diagnosis, intracranial, primary, spinal, metastasis, report, brain, lesion, fluid, csf, present, clinical |
| 6 | patient, month, median, survival, stage, cancer, year, bm, disease, lung, nsclc, metastasis, treatment, follow, recurrence, non, cell, treat, brain, receive |
| 7 | melanoma, patient, metastasis, brain, immunotherapy, therapy, braf, immune, metastatic, treatment, checkpoint, cell, inhibitor, mutation, disease, survival, carcinoma, clinical, response, treata |
| 8 | cancer, treatment, brain, therapy, drug, inhibitor, target, advisory, clinical, resistance, cell, agent, use, patient, research, blood, generation, kinase, therapeutic, barrier |
| 9 | srs, patient, brain, metastasis, month, radiosurgery, median, treat, stereotactic, lesion, local, control, tumor, gy, treatment, follow, radiation, volume, survival, wbrt |
| 10 | breast, cancer, medulloblastoma, woman, patient, metastatic, metastasis, negative, bone, treatment, invasive, tps, tumor, ductal, sp, node, positive, mastectomy, fgfr, triple |
| **Since 2020** | |
| 1 | patient, brain, metastasis, survival, month, treatment, cancer, group, median, lung, study, year, analysis, receive, method, age, therapy, diagnosis, os, overall |
| 2 | metastasis, brain, breast, cancer, tumor, cell, metastatic, patient, primary, high, use, study, expression, cd, gene, associate, result, negative, analysis, triple |
| 3 | brain, metastatic, patient, metastasis, cell, treatment, lung, case, present, cancer, report, year, therapy, immune, disease, cerebral, carcinoma, rare, clinical, associate |
| 4 | alk, brain, lung, metastasis, metastatic, cell, cancer, patient, tumor, lymphoma, crizotinib, alectinib, positive, disease, lorlatinib, anaplastic, kinase, use, treatment, rearrange |
| 5 | metastasis, patient, brain, melanoma, treatment, cancer, tumor, month, pet, case, year, clinical, survival, therapy, lung, disease, metastatic, primary, median, cell |
| 6 | brain, patient, metastasis, treatment, use, tumor, therapy, method, value, ici, imaging, qol, result, include, change, edema, clinical, sa, lesion, study |
| 7 | pt, patient, advisory, cancer, therapy, ipi, research, institution, metastasis, consultancy, funding, metastatic, grant, dose, median, breast, study, clinical, awake, brain |
| 8 | cell, cancer, expression, gene, target, tumor, beta, signal, brain, metastasis, pathway, resistance, mir, mechanism, role, therapeutic, alpha, inhibitor, proliferation, sample |
| 9 | patient, metastasis, brain, egfr, nsclc, mutation, treatment, cancer, month, study, lung, survival, non, group, treat, clinical, tki, cell, ci, result |
| 10 | patient, disease, metastasis, ctdna, treatment, cancer, clinical, therapy, brain, choriocarcinoma, pregnancy, present, trophoblastic, gestational, management, review, lmd, systemic, include, metastatic |
